# Supplementary material for: Association of Psychosocial and Health Factors with Long COVID Symptoms in Students in Medicine-Related Departments: A Cross-Sectional Survey
Source: Healthcare (Basel). 2025 Jul 30;13(15):1855. doi: 10.3390/healthcare13151855 (PMC12345682; doi:10.3390/healthcare13151855)
Supplement: Supplementary file 1 [file healthcare-13-01855-s001.zip › healthcare-3707168-supplementary.pdf]

Physical and Mental Health during the COVID-19 Pandemic Questionnaire

This questionnaire is designed to assess your personal health status during the COVID-19 pandemic. Your student ID will be used to match your responses with COVID-19 diagnosis history recorded at the school health center. After matching, the student ID will be deleted, leaving only an assigned ID for record-keeping. All personal information will remain strictly confidential. Please answer the following questions accordingly.

1. Demographic Inventory

Student ID: ____________.

Age: ________. Height:__________ cm. Weight:__________ kg.

Gender: □ Male □ Female □ Other

First Dose of Vaccine: □ None □ AZ □ Moderna □ BNT □ Medigen □ Other

Second Dose of Vaccine: □ None □ AZ □ Moderna □ BNT □ Medigen □ Other

Third Dose of Vaccine: □ None □ AZ □ Moderna □ BNT □ Medigen □ Other

COVID-19 Diagnosis: □ No □ Yes, Date of Diagnosis: ___________.

If answered "YES" to the previous question, then: □ Took NRICM101 □ Did not take NRICM101.

2. Health Status Checklist for the Past Month

(1) Have you experienced any of the following physical symptoms in the past month? Please mark "V" for the appropriate option in the following items. (multiple selections allowed)

□ No physical problems; □ Cold/respiratory symptoms; □ Fever;

□ Hypertension; □ Headache/dizziness/head pressure;

□ Skin redness/itching/atopic dermatitis/eczema/urticarial; □ Difficulty breathing;

□ Chest pain/tightness; □ Heart palpitations; □ Musculoskeletal or joint pain;

□ Hair loss; □ Oral ulcers; □ Impairment of smell or taste;

□ Frequent diarrhea; □ Decreased physical energy; □ Weight gain;

□ Rapid weight loss ；
□ Changes in menstrual cycle；
□ Fatigue.

(2) Have you experienced any of the following cognitive function conditions in the past month? Please mark "V" for the appropriate option in the following items. (multiple selections allowed)

□ Difficulty concentrating；
□ Short-term memory decline；
□ Spatial orientation problems；
□ Reduced comprehension ability.

(3) Have you experienced any of the following mental or psychological symptoms in the past month? Please mark "V" for the appropriate option in the following items. (multiple selections allowed)

□ No mental issues；
□ Anxiety；
□ Perceived high stress；
□ Mood instability；
□ Unexplained low mood；
□ Lack of motivation；
□ Depression；
□ Feelings of worthlessness or guilt；
□ Suicidal thoughts；
□ Persistent negative self-perceptions.

(4) Sleep Patterns in the past month (multiple selections allowed)

□ No problems in sleep;
□ Difficulty falling asleep (taking at least 30 minutes to fall asleep);
□ Difficulty maintaining sleep (waking up 2 or more times during the night);
□ Feeling fatigued despite adequate sleep;
□ Taking medication for sleep.

(5) Activity engagement in the past month (single choice)

□ No exercise routine;

□ Difficulty falling asleep (taking at least 30 minutes to fall asleep);

□ Regular ≥ 30-minute sweating exercise 3-5 times per week;

□ Regular ≥ 30-minute sweating exercise 1-2 times per week;

□ Occasional light activity (less than 30 minutes per session);

□ Significantly reduced outdoor activities compared to pre-pandemic levels.

(6) Changes in interpersonal relationships (single choice):

□ No change； □ Better； □ Worse.
